# Insight into the HIV-1 Vif SOCS-box–ElonginBC interaction

**DOI:** 10.1098/rsob.130100

**Published:** 2013-11

**Authors:** Zhisheng Lu, Julien R. C. Bergeron, R. Andrew Atkinson, Torsten Schaller, Dennis A. Veselkov, Alain Oregioni, Yi Yang, Stephen J. Matthews, Michael H. Malim, Mark R. Sanderson

**Affiliations:** 1 Randall Division of Cell and Molecular Biophysics, King's College London, 3rd Floor, New Hunt's House, Guy's Campus, London Bridge, London SE1 1UL, UK; 2 Department of Infectious Diseases, King's College London, Guy's Hospital, London SE1 9RT, UK; 3 Division of Molecular Biosciences, Department of Life Sciences, Imperial College London, South Kensington Campus, London SW7 2AZ, UK; 4 MRC Biomedical NMR Centre, National Institute for Medical Research, The Ridgeway, Mill Hill, London NW7 1AA, UK

**Keywords:** HIV-1 viral infectivity factor, SOCS-box domain, ElonginBC, NMR, solution structure

## Abstract

The HIV-1 viral infectivity factor (Vif) neutralizes cell-encoded antiviral APOBEC3 proteins by recruiting a cellular ElonginB (EloB)/ElonginC (EloC)/Cullin5-containing ubiquitin ligase complex, resulting in APOBEC3 ubiquitination and proteolysis. The suppressors-of-cytokine-signalling-like domain (SOCS-box) of HIV-1 Vif is essential for E3 ligase engagement, and contains a BC box as well as an unusual proline-rich motif. Here, we report the NMR solution structure of the Vif SOCS–ElonginBC (EloBC) complex. In contrast to SOCS-boxes described in other proteins, the HIV-1 Vif SOCS-box contains only one α-helical domain followed by a β-sheet fold. The SOCS-box of Vif binds primarily to EloC by hydrophobic interactions. The functionally essential proline-rich motif mediates a direct but weak interaction with residues 101–104 of EloB, inducing a conformational change from an unstructured state to a structured state. The structure of the complex and biophysical studies provide detailed insight into the function of Vif's proline-rich motif and reveal novel dynamic information on the Vif–EloBC interaction.

## 2. Introduction

The apolipoprotein B mRNA-editing enzyme, catalytic polypeptide-like 3 (APOBEC3, A3) family of cytidine deaminases contains seven members named from A to H [1] that are constitutively present in cells and exhibit diverse physiological functions [2–4]. APOBEC3G (A3G) appears to have the most active antiviral phenotype for HIV-1, as recently described in several reviews [5–8].

The viral infectivity factor (Vif) protein of HIV-1 is an accessory protein required for virus replication in natural targets of infection [5]. Vif functions by counteracting the APOBEC3-mediated pathway of intrinsic antiviral defence by inducing the proteasomal degradation of APOBEC3 proteins, including A3DE [9], A3F [10–12], A3G [13–17] and some A3H proteins [18–20]. Degradation is achieved through the formation of an E3 ubiquitin ligase core complex composed of ElonginB (EloB), ElonginC (EloC), Cullin5 (Cul5) and RING-box protein 2 [21–25] in the presence of Vif. The core binding factor β (CBFβ), a newly discovered chaperone, is crucial for the folding and stabilizing Vif [26–28], the Vif–APOBEC3G interaction, [29,30] and regulation of host gene expression [28]. The formation of this E3 ligase complex results in ubiquitination of APOBEC3 proteins, and therefore neutralization of APOBEC-mediated antiviral activity. The interface afforded by the interaction of Vif with the cellular factors mentioned above is widely viewed as a potential target for the development of new anti-HIV drugs [5,6].

Vif possesses various motifs that bind these cellular factors that have been linked to direct interactions with the proteins mentioned above. The N-terminal half of Vif mediates the interaction with members of the APOBEC3 family [31,32]. Recent work from John Gross's group shows that the N-terminal residues 1–140 also interact with CBFβ [28]. In the middle of the Vif sequence, a zinc-binding HCCH motif has been proposed to interact with Cul5 [33–35]. C-terminal to this HCCH domain, the suppressors-of-cytokine-signalling-like domain (SOCS-box) of Vif binds to the EloB–EloC heterodimer (EloBC) along with Cul5 [22,36–38]. The SOCS-box contains a conserved SLQYLA motif (residues 144–149), called the BC-box, which interacts with EloBC. Mutation of this motif leads to the inactivation of Vif, indicating an essential role for this domain [14,22,36,39,40]. The SOCS-box also has a critical proline-rich motif (161PPLPS165, PPLPS motif) [41,42] downstream of the BC-box, whose molecular mechanism of action remains unclear. For instance, some reports suggest a role in Vif homo-multimerization [43], whereas biophysical studies indicate that the proline-rich motif interacts with the flexible C-terminus of EloB and is not required for the oligomerization [37,44].

Owing to the difficulty in overexpressing, purifying and crystallizing full-length folded soluble Vif and APOBEC3G [45,46], it remains unclear from a structural biological perspective how Vif recruits cellular factors and interacts with APOBEC3G in order to stimulate ubiquitination. Currently, the crystal structure of BC-box has been solved, but not that of the entire SOCS domain [38]. We have therefore employed a range of NMR techniques to dissect the structure and dynamics of the Vif SOCS–EloBC complex. In particular, Vif SOCS-box, EloC and EloB all experience structural changes during the SOCS–EloBC interaction, as proposed previously [37]. The solution structure of EloBC was solved by NMR in 2008 [47] in complex with the BC-box of SOCS3. Here, we present a solution structure of the Vif SOCS–EloB–EloC heterotrimer. The structure of the complex is calculated by HADDOCK combined with long-distance restraints from paramagnetic relaxation enhancement (PRE) experiments and NMR chemical shift perturbations. We find that the van der Waals surface calculated around the PPLPS motif of SOCS-box touches the van der Waals surface calculated about the EloB C-terminus, thereby supplementing the tight binding interface between the BC-box and EloC. Upon binding, the C-terminal tail of EloB experiences a structural change from a flexible state to a partially folded state. Consistent with earlier structure–function analyses, the leucine, the third proline and the adjacent serine are the most important residues in this motif [40].

## 3. Material and methods

### 3.1. Protein expression and purification

The SOCS–EloBC complex for NMR spectroscopy was prepared as described previously [37]. Essentially, EloBC dimer and SOCS-box peptide were expressed individually in the *Escherichia coli* BL21 (DE3) Rosetta strain in LB media or M9 minimal media supplemented with different isotopes (^13^C, ^15^N, ^2^H), depending on the experiments. EloBC was purified in 20 mM Tris buffer pH 7.0, 50 mM NaCl and solubility-enhancement-tagged SOCS-box peptide was purified in 20 mM Tris buffer pH 8.0, 500 mM NaCl. They were mixed at a 1 : 1 ratio after elution from the Ni-NTA column and loaded onto a Superdex 75 gel filtration column to remove unbound components. EloBC-labelled sample or SOCS-labelled sample was then used in NMR spectroscopy experiments.

### 3.2. NMR spectroscopy

NMR spectra were acquired at 25°C on Varian NMR 800 MHz and Bruker Avance 700 MHz spectrometers equipped with 5 mm triple-resonance *z*-axis gradient cryogenic probes. A 0.2 mM sample (550 μl) was prepared in 50 mM phosphate buffer pH 7.0, 10% D_2_O, 0.05% sodium azide with protease inhibitors (Roche) tablet. HNCO, CBCA(CO)NH, HNCA, HNCACB, HN(CA)CO, HN(CA)HA, HCCH-TOCSY, ^1^H-^15^N TROSY-HSQC and ^13^C-edited NOESY-HSQC were recorded on EloBC, and HNCO, HN(CA)CO, HNCA, HNCACB, CBCA(CO)NH, HN(CA)HA, HCCH-COSY, HCCH-TOCSY, ^15^N-edited NOESY-HSQC, ^13^C-edited NOESY-HSQC, ^1^H-^15^N HSQC and ^1^H-^13^C HSQC spectra were used for the SOCS-box peptide backbone and side-chain assignment. All the spectra were processed with NMRPipe [48] and analysed with CcpNmr suite [49,50]. As for the perturbation studies, each EloBC mutant sample was prepared in the same way as wild-type and divided into two aliquots. Purified SOCS-box peptide was added to one aliquot at a 1.2 : 1 ratio and the other was made up to the same volume as the first aliquot with NMR buffer. Relaxation experiments were recorded on the unbound SOCS-box peptide at 25°C at 500 and 700 MHz magnetic field, respectively.

### 3.3. Paramagnetic relaxation enhancement experiments

Residues G143, Q158 and R167 on SOCS-box peptide were mutated to Cys, for use in paramagnetic labelling studies. Mutant protein was mixed with an approximately fivefold excess of dithiothreitol (DTT) for 2 h after elution from the Ni-NTA column. This was followed by separation of excess DTT by gel filtration chromatography. The SOCS-box monomer sample from the size exclusion column was collected and incubated with either the diamagnetic (1-acetyl-2,2,5,5-tetramethyl-*Δ*3-pyrroline-3-methyl) methanethiosulfonate or the paramagnetic (1-oxyl-2,2,5,5-tetramethyl-*Δ*3-pyrroline-methyl) methanethiosulfonate (Toronto Research Chemicals) overnight at 4°C. Each modified SOCS-box sample was dialysed against NMR buffer and mixed with ^15^N-labelled EloBC in NMR buffer at a 1.1 : 1 ratio. The mixed sample was then used for NMR spectral acquisition. A ^1^H-^15^N HSQC spectrum was recorded for each 50 μM sample with a 3-h acquisition. Intensity ratios were converted to distances according to an established method [51] by using the Solomon–Bloembergen equation [52]. Because of the dynamics of the labels, the diameter of the paramagnetic molecule was added to or subtracted from the calculated distance, thus obtaining the upper or the lower limitation distance between the Cys and observed ^15^N-labelled EloBC residues.

### 3.4. Structure determination

SOCS-box peptide structures in the bound state were generated by Chemical Shift ROSETTA (CS-ROSETTA) [53] by inputting NOE data and chemical shift values into the BMRB CS-ROSETTA server (condor.bmrb.wisc.edu/bbee/rosetta/). Structures were further refined according to the Rosetta refinement protocol [54]. EloB and EloC structures were generated *de novo* as well as on the server. Using NMR perturbation studies based on ^1^H-^15^N HSQC spectra and PRE data that provide semi-quantitative long-distance constraints, the HADDOCK approach was adopted for the structure calculation of the complex [55]. In our previous work, it has been proved by various biophysical assays that the EloB DVMK stretch interacts with the proline-rich motif [37], so in the calculation on the WeNMR web server [56], five residues in SOCS-box (Q146, A149, L163, P164 and S165), four residues in EloB (D101, V102, M103 and K104) and two residues in EloC (A82 and L86) were selected as active residues. The interfacial residues sitting between the SOCS-box proline-rich motif and the C-terminus of EloB were allowed to fully move at all stages. A file with distance restraints that are always enforced was provided. Two thousand initial complex structures were generated and the best 200 structures were chosen for explicit solvent refinement. The clustering cut-off is set to 5 Å, four structures per cluster. Default parameters excluding the settings above were always applied. The assignments and structures have been deposited to BMRB (ID 19333) and PDB (ID 2MA9), respectively.

### 3.5. ITC binding assays

EloBC dimer sample and SOCS-box peptide were concentrated to 0.2 and 0.02 mM, respectively. All samples were dialysed against binding buffer with 20 mM Tris pH 7.5, 250 mM NaCl and 0.05% sodium azide. ITC was performed on an ITC200 calorimeter (MicroCal, Northampton, MA). Titrations were conducted by injecting 20 aliquots of 2 μl of EloBC sample into cells containing SOCS-box peptide sample at 25°C. Fresh samples were prepared thrice in order to record ITC experiments in triplicate, and one typical set of results is presented.

## 4. Results

### 4.1. The flexibility of the unbound SOCS-box domain

**Figure 1.**
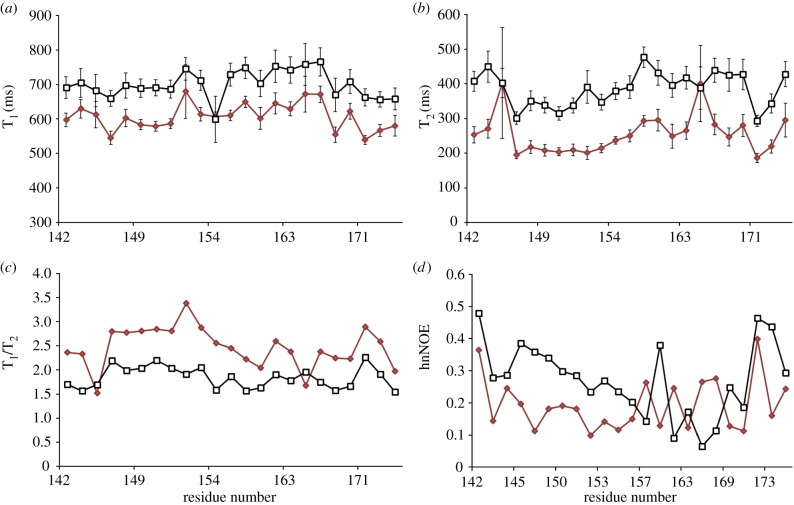
^15^N relaxation rate constants at 500 MHz (red lines) and 700 MHz (black lines) magnetic fields (*a*) T_1_, (*b*) T_2_, (*c*) T_1_/T_2_ and (*d*) hnNOE plotted against residue number.

In order to address the challenges associated with Vif insolubility, we N-terminally fused the Vif SOCS-box to a solubility-enhancement tag that does not increase the molecular weight substantially and therefore is suitable for NMR studies [57]. In previous work, it was found that the unbound SOCS-box lacks secondary structure [37]. Here, the NMR relaxation experiments were recorded at two magnetic field strengths (11.75 and 16.4 T, 500 and 700 MHz at ^1^H frequency) in order to observe the flexibility of the SOCS-box peptide. The T_1_, T_2_, T_1_/T_2_ ratio and ^15^N heteronuclear nuclear Overhauser effect (hnNOE) are plotted against the residue numbers (figure 1). The fact that the T_1_ values of BC-box are consistently the same over the span of residues 144–154 indicates that this region is less dynamic and tumbles isotropically compared with the rest residues of the SOCS-box. However, it is of note that the N-terminal-fused tag attached to this region may also contribute to its limited motion. T_2_ values suggest the existence of fast motion. In addition, the variable low values of hnNOE reveal that the SOCS-box peptide possesses considerable internal motion, especially the region following the BC-box. These relaxation results show that the SOCS-box has a random coil conformation before binding to EloBC.

### 4.2. The structure of SOCS-box domain

**Figure 2.**
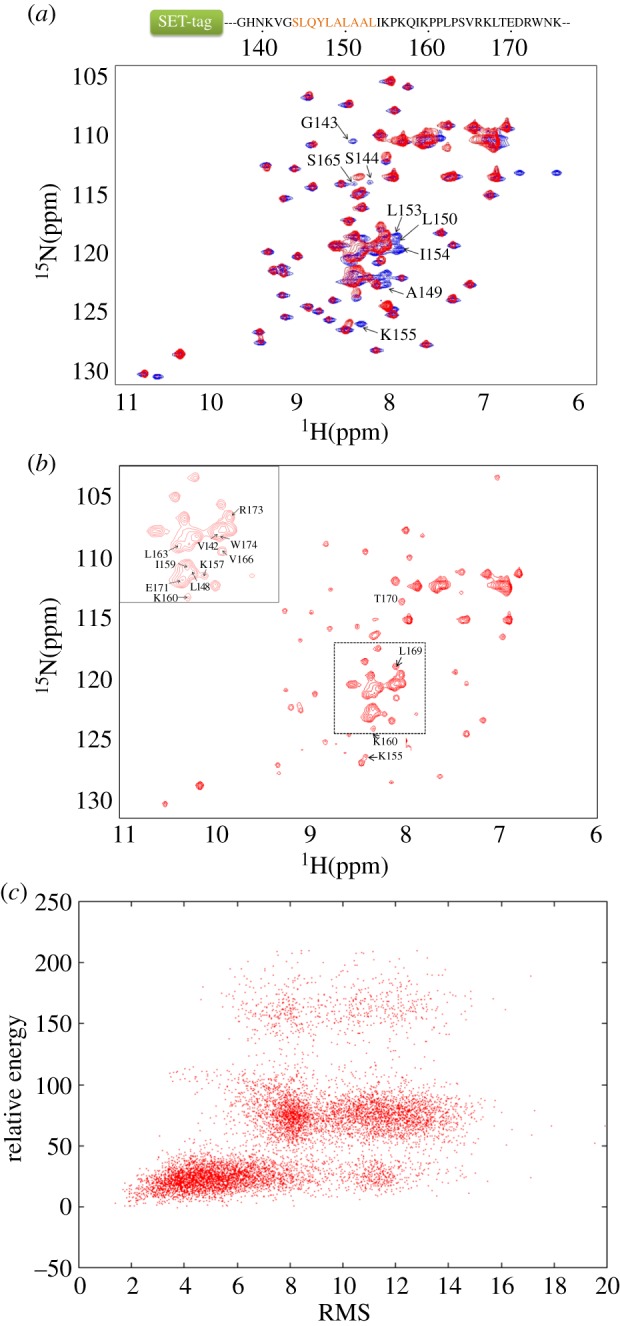
^1^H–^15^N HSQC spectra of Vif SOCS-box protein, with the sequence of the SOCS-box peptide shown on top. The 56-residue SET-tag is simplified with a rectangle, followed by the SOCS-domain peptide. The BC-box is highlighted in orange. (*a*) The overlay of two spectra recorded on the SOCS-box peptide in the free state (blue) and in the complex with EloBC (red). (*b*) Details of the spectrum recorded on the bound SOCS-box peptide. The peaks assigned to residues corresponding to the Vif SOCS-box are indicated. The rest of the peaks distributing in the spectrum are from the SET-tag. (*c*) The Rosetta score versus RMSD plot from CS-Rosetta structural calculation protocol. A total of 10 000 structures were generated. (*a*,*b*) are reproduced with permission from [37].

**Figure 3.**
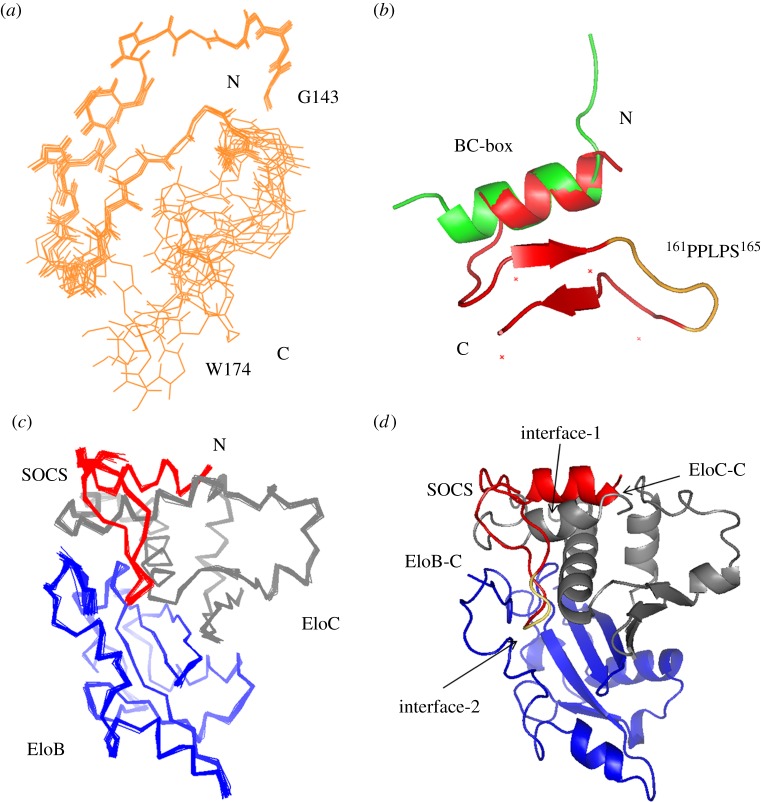
The structures of the HIV-1 Vif SOCS peptide and the SOCS–EloBC complex. (*a*) SOCS peptide alignment. Overlap of ten backbone structures for the SOCS-box after Rosetta refinement. (*b*) Structural comparison with the SOCS-box crystal structure (3DCG, chain E). Peptide structure is aligned against the crystal structure in green. The 165PLS167 sequence is highlighted in yellow. (*c*) 20 structures of the SOCS–EloBC complex with the lowest energy are aligned after HADDOCK refinement. (*d*) Ribbon representation of the complex. The Vif SOCS peptide is coloured in red. EloB and EloC are in blue and grey, respectively. The 165PLS167 region of SOCS is highlighted in yellow.

In order to solve the structure of SOCS-box, the NMR spectra (figure 2*a,b*) [37] were recorded on the labelled peptide produced from *E. coli* in the unbound state and in the complex with unlabelled EloBC heterodimer, for which the two components were co-expressed and co-purified. Although comprehensive NOE data are not available for the entire SOCS-box α-helical region, the 32-amino-acid peptide in the complex adopts a well-defined structure based on chemical shift analysis using CS-Rosetta combined with a limited set of NOE measurements (figure 2*c* and table 1). The RMSD between the lowest energy structure and the helical BC-box crystal structure (PDB ID: 3DCG, chain E) [38] is 0.43 Å after refinement by the Rosetta protocol. The final structure has an αββ structure in which the two β-strand-like elements are connected by the proline-rich motif loop (figure 3*a,b*), resulting in an exposed loop that projects into solution and is accessible for interaction with other molecules. The second β-strand appears to be flexible compared with the other regions.

**Table 1.** Structural statistics for the bound SOCS-box peptide from CS-Rosetta.

NOE restraints comprised in calculations	
short range	59
medium range	11
long range	8
average RMSDs from the mean structure	
backbone average (Å)	0.54 ± 0.19
heavy atom average (Å)	0.65 ± 0.22
Ramachandran plot	
most favoured region (%)	96.7
allowed region (%)	3.3
generously allowed region (%)	0
disallowed region (%)	0

### 4.3. The solution structural determination of the SOCS-box–Elongin BC complex

**Figure 4.**
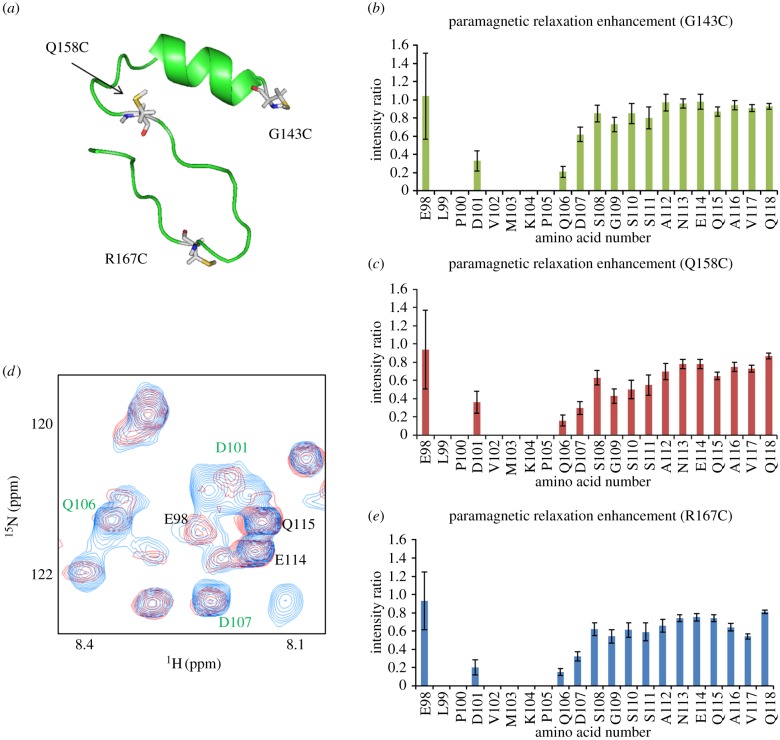
PRE experiments. (*a*) The three mutated sites in Vif which were distributed at widely separated positions in the peptide. G143C, Q158C and R167C are highlighted in white and the sulfur of the cysteine to be modified by label is coloured in yellow. Each predicted mutant structure is generated by Pymol in order to determine that the side chain of cysteine would not be buried inside the molecule. (*b*) Intensity ratio of EloB C-terminus for G143C mutant. (*c*) Intensity ratio of EloB C-terminus for Q158C mutant. (*d*) A portion of the ^1^H-^15^N HSQC spectra of EloBC in the presence of the paramagnetic molecule-labelled SOCS-box R167C peptide (red) or the diamagnetic molecule-labelled R167C peptide (blue). Peaks assigned to the EloB C-terminal tail are indicated in green (significantly broadened) or in black (not affected). (*e*) Intensity ratio of EloB C-terminus for R167C mutant. The intensity of peaks from residues 101–104 are broadened owing to the interaction regardless of the paramagnetic effect. The error bars show how the noise of the spectra affects the intensity ratio. Stronger peaks provide smaller deviation.

The binding of the HIV-1 Vif SOCS-box domain to EloBC forms a stable heterotrimer with a 1 : 1 : 1 stoichiometric ratio determined by gel filtration analysis [37]. In order to acquire additional intermolecular restraints among the three components, PRE experiments were performed [52]. As no cysteines are present in the SET-tag-fused SOCS-box peptide, single-point cysteine mutations could be generated in this peptide at selected locations to measure PREs within the other components of the complex, namely EloBC. In each molecule of the complex, a mutated SOCS-box peptide was engineered with a single cysteine substitution enabling the observed relaxation enhancements to be assigned to the paramagnetic moiety conjugated to the amino acid residue. Point mutations (G143C, Q158C and R167C) were placed at three widely separated sites in the SOCS peptide in order to increase the number and spread of intermolecular measurements (figure 4*a*). The binding of all mutants was checked by isothermal titration calorimetry (ITC) to verify that the mutation did not interfere with the binding affinity of SOCS–EloBC (see electronic supplementary material, figure S1). As the portion of the SOCS-box binding to EloBC dimer has been localized to the BC-box and the PPLPS motif, mutations were made outside these functional domains to minimize any artefactual interactions. In PRE experiments, the peak-intensity ratios measured in the EloB carboxyl terminus provide a clear indication that the DVMK stretch at the C-terminus of EloB is close in space to the labelled cysteines of the Vif SOCS-box (figure 4*b,c*), especially the R167C residue (figure 4*d,e*). The overlay of a 20-solution-structure alignment from the cluster is shown in figure 3. Structural statistics for the cluster of structures are listed in table 2.

**Table 2.** Structural statistics for the SOCS–EloBC complex.

HADDOCK cluster number produced	1
average RMSDs from the mean structure	
backbone average (Å)	0.26±0.03
heavy atom average (Å)	0.52±0.04
HADDOCK statistics	
van der Waals energy (kcal mol^-1^)	–63.5±2.4
electrostatic energy (kcal mol^−1^)	–574.5±15.9
desolvation energy (kcal mol^−1^)	3.1±3.3
restraints violation energy (kcal mol^−1^)	0.3±0.16
buried surface area (Å^2^)	2438.6±46.0
experimental PRE distance restraints	
number	58
distance violation (Å)	2.76±1.90
Ramachandran plot	
most favoured region (%)	82.3
allowed region (%)	15.2
generously allowed region (%)	1.4
disallowed region (%)	1.1
deviations from ideal geometry	
angles (degree)	0.6
bonds (Å)	0.003

**Figure 5.**
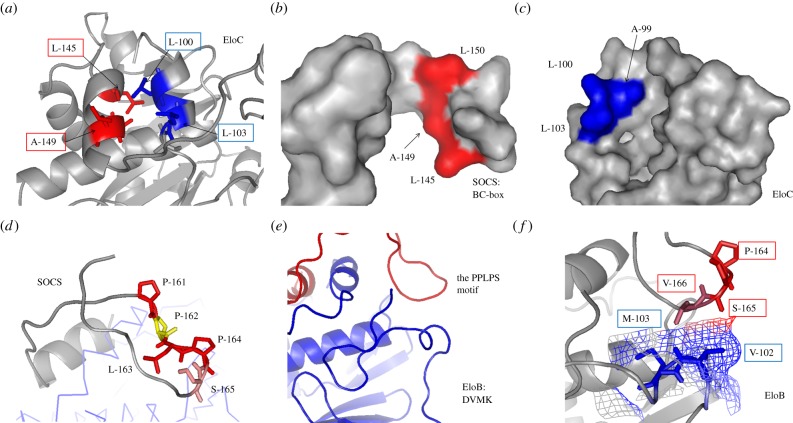
Interaction between the SOCS-box and EloBC. (*a*) The interface of the BC-box and EloC binding site. Active side-chains are presented and labelled. (*b*) The binding surface of BC-box. Resides are coloured in red. (*c*) The binding surface of EloC C-terminal α-helix. Resides on the interface are coloured in blue. (*d*) The proline-rich motif downstream from the SOCS-box is coloured. Residues are highlighted in various colours. The second proline (residue 162) in yellow is buried in the complex. (*e*) The C-terminus of EloB, including the DVMK stretch. Blue, EloB; red, SOCS Peptide. (*f*) The interface of EloB DVMK stretch and the Vif proline-rich motif. The van der Waals radii of V102 and M103 are presented. The interacted regions by the PPLPS motif are coloured differently.

The structural model provides confirmation that the SOCS-box crosses the EloC carboxyl α-helix and binds to the flexible EloB carboxyl terminus. Residues L145, A149, L150 of the Vif SOCS-box and A99, L103 of EloC bind to each other (figure 5*a*) by forming hydrophobic interfaces that drive the SOCS–EloC interactions (figure 5*b,c*) typical of a high-affinity protein complex [58,59]. Hydrophobic interaction is the major force driving the formation of biological complexes [22,39]. The structure of this portion of the complex very closely matches that of the crystal structure (PDB ID: 3DCG) [38]. The studies on the PPLPS motif show that 164PS165 plus V166 interacts with the EloB DVMK stretch rather than the entire PPLPS motif (figure 5*d,e*). This proline–serine loop is stabilized by an antiparallel β-sheet-like structure. Within EloB, the C-terminus is flexible in solution, as shown by the narrow NMR line widths for this region [37], whereas in the presence of SOCS-box, the DVMK stretch experiences a conformational change and becomes partially helical (figure 5*e*). The interface between 164PS165 and the DVMK stretch is formed by close spatial positioning of the residues and are bound by weak van der Waals forces (figure 5*f*), implying that this interaction may not be a strong interaction although it was observed from the NMR perturbation experiments and ITC studies of SOCS-box and its mutants [37].

### 4.4. The interaction between the PPLPS motif and the DVMK stretch

**Figure 6.**
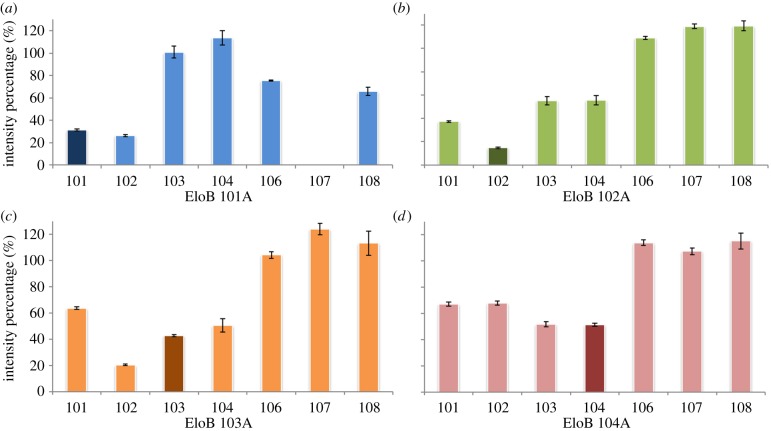
(*a*–*d*) The NMR perturbation studies on the Vif SOCS–EloBC complex. ^1^H–^15^N HSQC spectra were recorded on the labelled EloBC dimer in the absence and in the presence of SOCS-box peptide. The percentage represents the intensity ratio of bound peaks to unbound peaks. The numbers on the *x*-axis indicate the residues at the C-terminus of EloB.

In order to define further the SOCS–EloB interface, we subsequently mutated the four residues at the C-terminus of EloB, respectively, and measured NMR perturbations to the line widths of distal amino acids. Interestingly, peaks from this domain follow a distinct decrease in intensity irrespective of which residue is mutated (figure 6). As for the EloB-D101A mutant, A101 is still perturbed upon binding, although residues M103 and K104 are not impacted. The NMR spectrum recorded for this mutant displayed peak shifts for residues 103–118, suggesting a conformational disruption by this mutation (data not shown). Profiles from the other three mutants match the same profile as for wild-type [37], indicating that the interaction between the PPLPS motif and the C-terminus of EloB is not specifically driven by these side-chains within the DVMK stretch.

We then asked the question whether individual residues in the PPLPS motif contribute more to the interaction with EloB. Several single-point mutants in the motif were made and ITC was used to quantify the thermodynamics of the interaction. Although sequence analysis of Vif and EloB amino acids indicates that the PPLPS motif of HIV-1 Vif and residues 101–104 (DVMK stretch) of EloB are both highly conserved (see electronic supplementary material, figure S2 and table S1), results from ITC reveal that the mutation of any residue within the PPLPS motif does not affect the binding affinity regardless of the entropy change (table 3; electronic supplementary material, figure S3), which is thought to be caused by internal conformational changes owing to the single-site mutations [60]. It can therefore be concluded that the second interface between the PPLPS motif and the DVMK stretch is driven by weak van der Waals forces.

**Table 3.** Thermodynamic characterization of SOCS–EloBC interaction.

	*K*_d_ (μM)	*K*_a_ (μM^−1^)	*Δ*H_obs_ (kcal mol^–1^)	*Δ*G (kcal mol^–1^)	–T*Δ*S (kcal mol^–1^)
WT	1.225	0.82±0.06	−10.2±1.6	−7.9	2.3
SPLPS	2.14	0.52±0.18	−8.3±2.3	−7.6	0.7
PSLPS	1.24	0.86±0.24	−8.8±2.0	−7.9	0.9
PPLSS	1.36	0.77±0.19	−10.7±2.8	−7.8	2.9
PPLPA	1.18	0.89±0.24	−8.6±0.3	−7.9	0.7
APLAS	1.10	0.97±0.28	−8.8±0.2	−8.0	0.8
AAAPS	1.50	0.68±0.11	−9.8±1.7	−7.9	1.9

## 5. Discussion

**Figure 7.**
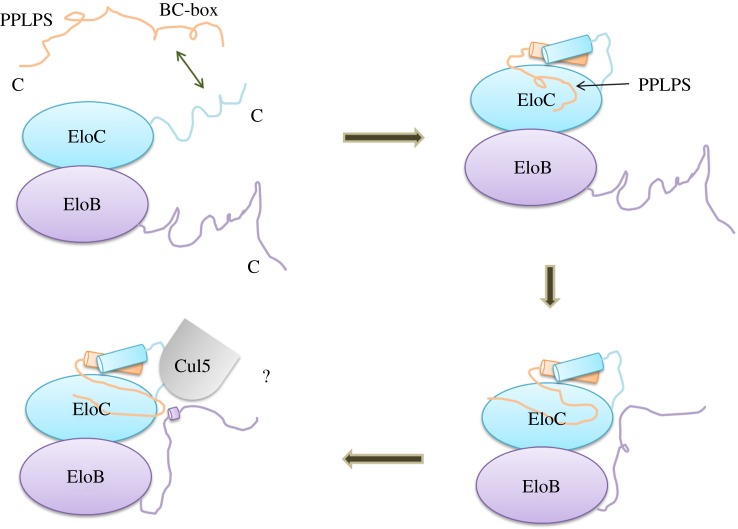
Schematic of the proposed induced-folding mechanism. The SOCS-box peptide includes an α-helix domain (represented as SLQ) and a proline-rich domain (represented as PPLP). The formation of the Vif SOCS–EloBC complex is mainly driven by hydrophobic interactions between Vif BC-box and EloC C-terminus via a conformational change process. Then, the proline-rich motif induces the EloB C-terminal tail to fold, forming a common interface to recruit cellular factors, perhaps Cul5. The various interaction events are presented in succession. The C-terminus of each subunit is indicated.

Here, we present the first structure of the HIV-1 Vif SOCS–EloBC complex including the conserved proline-rich motif of Vif. Interestingly, the side-chains of P161, L163 and P164 are exposed in the solution, whereas the side-chain of the second proline (P162) is buried within the protein, suggesting that this proline is less important compared with the other residues. This result agrees with previous work showing that mutations at the first and third proline decrease Vif function in cells and result in a lack of formation of the ubiquitination complex [37,40]. In our previous study [37], we reported that in the absence of SOCS-box the carboxyl terminus of EloB is flexible in solution and that the binding to SOCS-box induces structural changes in the disordered DVMK stretch according to the T_1_/T_2_ relaxation ratio [37]. This small helix in the EloB C-terminus is also observed in the SOCS2–EloBC complex [61]. Therefore, it can be concluded that the DVMK stretch forms a helix upon binding to SOCS-box. Considering the weak binding between the PPLPS motif and the DVMK stretch, which is not essential for SOCS–EloBC binding, though it is required for HIV-1 Vif function in cells, we further suggest that it is the α-helix of EloC that first drives the induced folding of Vif, followed by the interaction between the PPLPS motif and EloB. The final interface formed by the PPLPS motif and the induced folding of the DVMK stretch may be required to form an E3 ubiquitin ligase complex, perhaps specifically to recruit Cul5 (figure 7).

As we have demonstrated here, the interaction between Vif's PPLPS motif and EloB's DVMK has low affinity and is mainly a weak van der Waals interaction. We also show that it is coupled with significant structural rearrangements in both Vif and EloB. Yet it is a critical interaction for the recruitment of a functional E3 ubiquitin ligase, CBFβ binding [62] and degradation of A3G *in vivo* [63]. It is therefore tempting to speculate that this interaction could be targeted by small molecule inhibitors for the design of a new class of anti-HIV drugs. Furthermore, the NMR spectra reported here could form the basis for an assay to screen small molecules libraries. Yet, as the interaction is based on flexible regions of these proteins, there is presumably no obvious binding pocket for a small molecule to bind to. In that regard, a small peptide inhibitor, mimicking the PPLPS motif engaging EloB upon Vif binding, would be a more likely candidate for interrupting this interaction.

**Figure 8.**
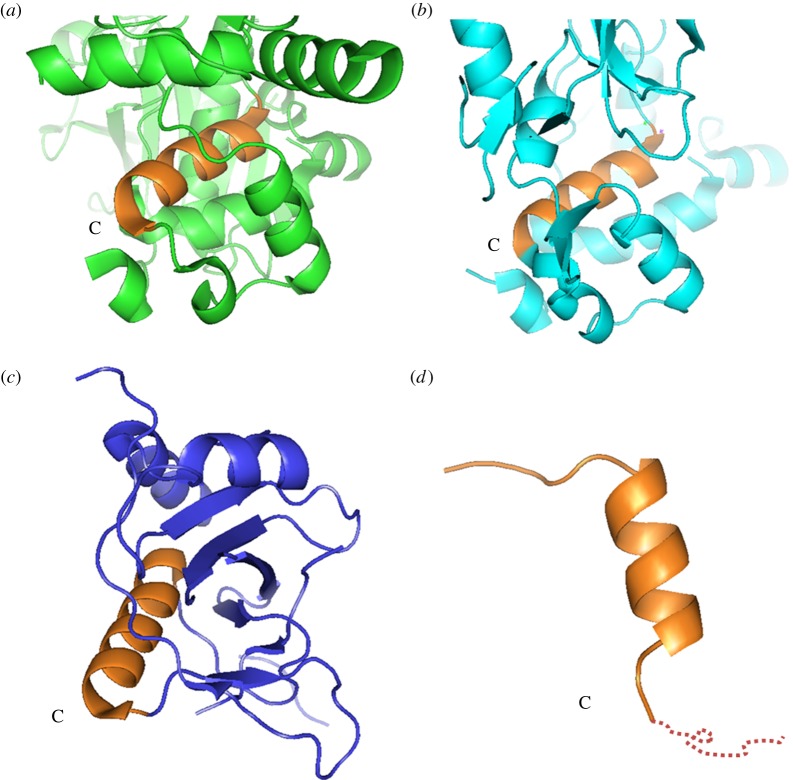
Structures of SOCS family proteins. Close-up of the SOCS domains from published structures of different SOCS families. The C-terminus of each SOCS α-helical domain is labelled. (*a*) SOCS2 domain (2C9W). (*b*) SOCS4 domain (2IZV). (*c*) SOCS6 domain (2VIF). (*d*) SOCS3 domain (3DCG). The unobserved downstream sequence of SOCS3 is presented by a dotted line. The SOCS domain on each structure is shown in brown.

The SOCS family (SOCS1–7) proteins are indispensable regulators, functioning in many pathways, including ubiquitination and transcription, and have high sequence similarity. Published structures show that they all share a common α-helical structure. It is of note, however, that the downstream sequences have a different spatial portfolio depending on the structural family. Domains from SOCS2 and SOCS4 (PDB ID: 2C9W, 2IZV) contain three small α-helixes that are also known to interact with the EloB C-terminus (figure 8*a,b*) [61,64], whereas SOCS6 (PDB ID: 2VIF) adopts a partially folded structure (figure 8*c*), and the SOCS3 (PDB ID: 3DCG) downstream sequence cannot be identified in the X-ray crystallographic analysis (figure 8*d*) [38,65], which suggests that the downstream structure is flexible in solution and can be only observed by NMR. Interestingly, the SOCS-boxes of Vif proteins from other retroviruses, such as HIV-2, simian immunodeficiency virus from mandrill (SIV_MND_) or bovine immunodeficiency virus (BIV), contain the BC-box, but not a proline-rich motif [63,66,67]. Our results report, for the first time, structural insight into the whole HIV-1 Vif SOCS domain, including a BC-box and the proline-rich motif in the presence of EloBC, and its dynamic behaviour in the SOCS–EloBC interaction.

## Supplementary Material

Figure S1. ITC studies on the Cysteine mutants

## Supplementary Material

Figure S2. Amino acid sequence alignments of Vif and EloB

## Supplementary Material

Figure S3. ITC raw data of the SOCS-EloBC binding studies

## Supplementary Material

Table S1

## Data accessibility

The DNA accession information is listed in the electronic supplementary material, table S1.
