# Supplementary figures and images for: Insight into the HIV-1 Vif SOCS-box–ElonginBC interaction
Source: Open Biol. 2013 Nov;3(11):130100. doi: 10.1098/rsob.130100 (PMC3843819; doi:10.1098/rsob.130100)

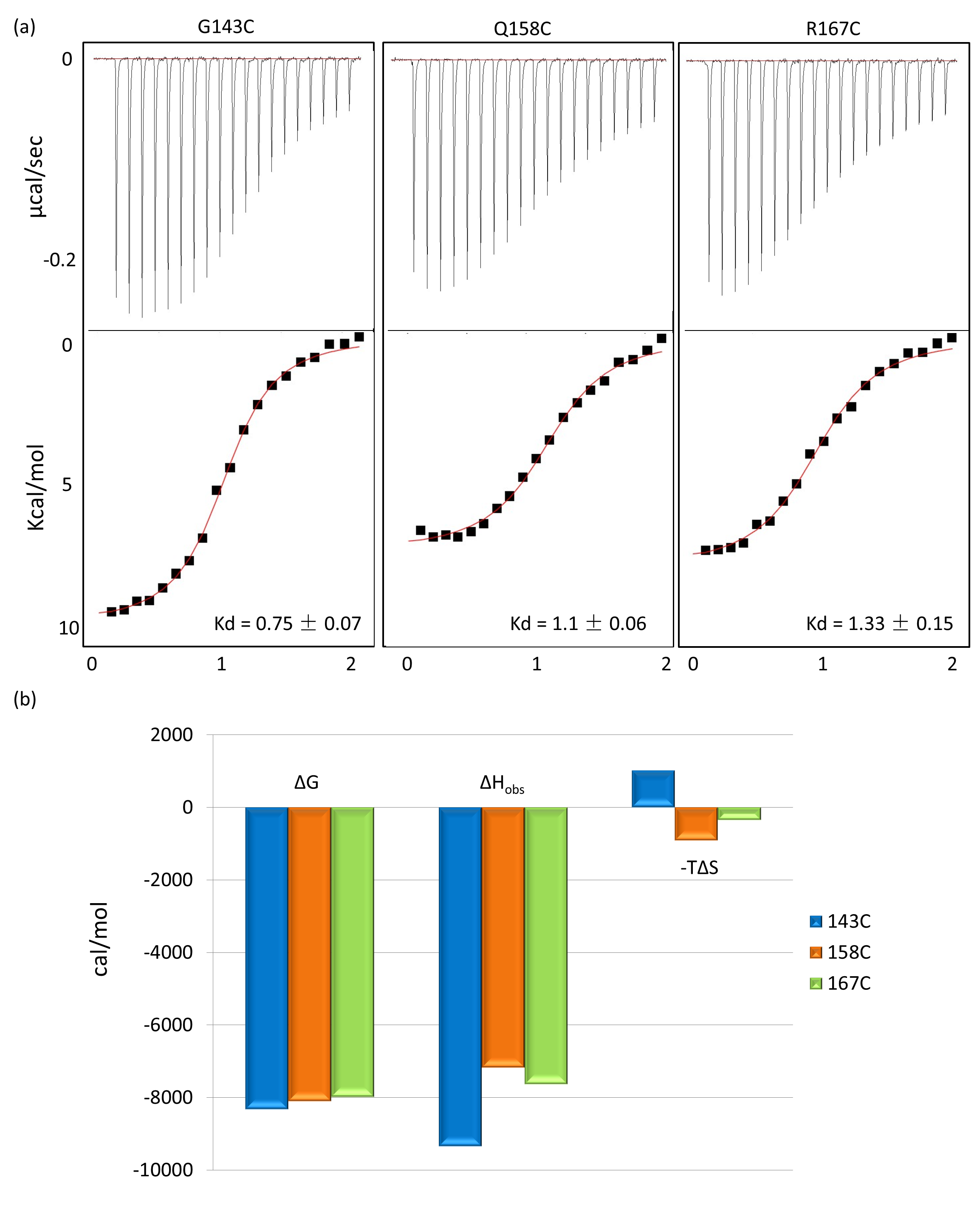

Supplement: Figure S1. ITC studies on the Cysteine mutants [file rsob130100supp1.tif]

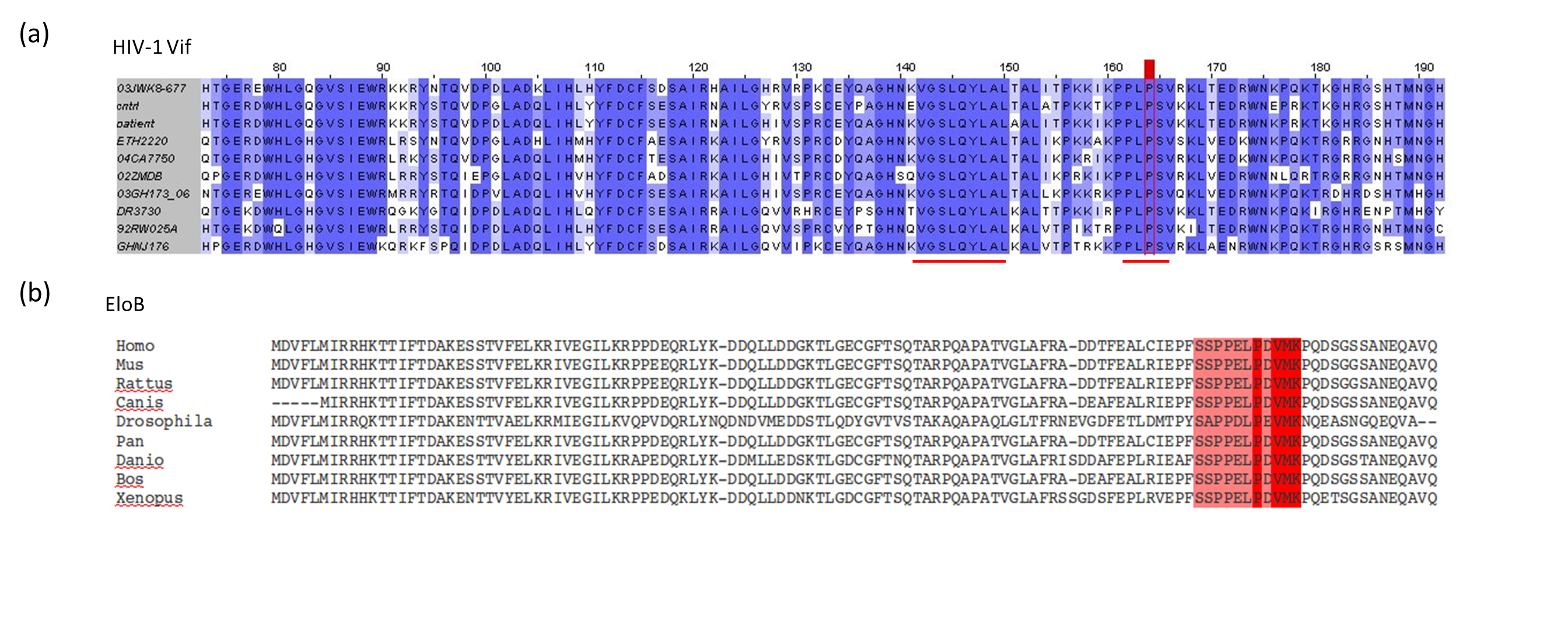

Supplement: Figure S2. Amino acid sequence alignments of Vif and EloB [file rsob130100supp2.tif]

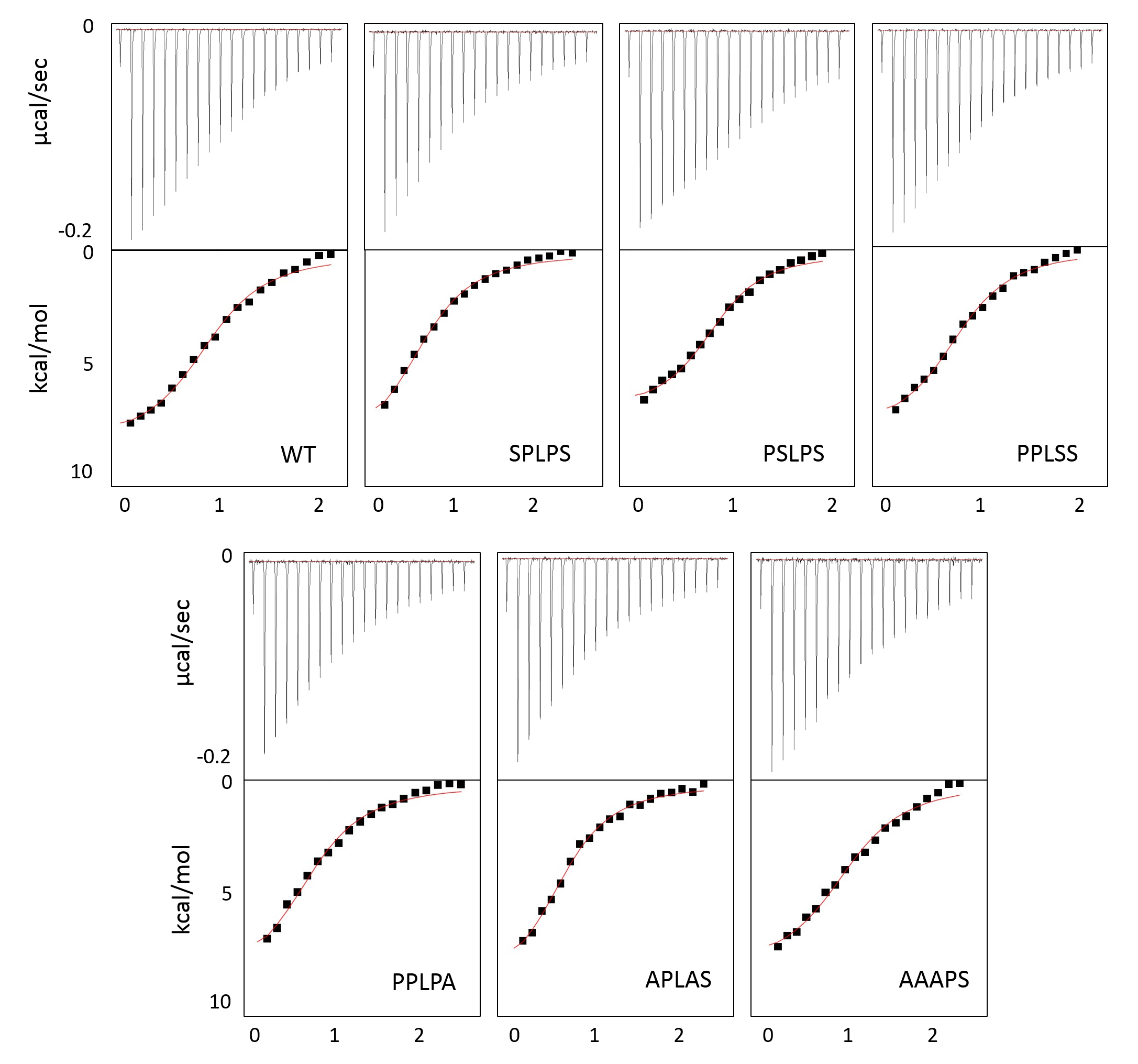

Supplement: Figure S3. ITC raw data of the SOCS-EloBC binding studies [file rsob130100supp3.tif]
