# Supplementary material for: Insight into the HIV-1 Vif SOCS-box–ElonginBC interaction
Source: Open Biol. 2013 Nov;3(11):130100. doi: 10.1098/rsob.130100 (PMC3843819; doi:10.1098/rsob.130100)
Supplement: Table S1 [file rsob130100supp4.doc]

Table S1. The authoritative source & Accession Numbers of sequences in alignment

| **Species / Name** | **Authoritative source** | **Accession Number** |
| --- | --- | --- |
| *Vif amino acid sequence* | | |
| 03JWK8-677 | GenBank | AAS93516.1 |
| ontrl | GenBank | AAA79546.1 |
| oatient | GenBank | AAA79646.1 |
| ETH2220 | GenBank | AAB36502.1 |
| 04CA7750 | GenBank | ABX61044.1 |
| 02ZMDB | GenBank | BAF33217.2 |
| 03GH173_06 | GenBank | BAF42372.1 |
| DR3730 | GenBank | BAF32554.1 |
| 92RW025A | GenBank | BAF42516.1 |
| GHNJ176 | GenBank | BAE95928.1 |
| *EloB amino acid sequence* | | |
| Homo | GenBank | AAC08452.1 |
| Mus | GenBank | NP_080581.1 |
| Rattus | NCBI ID | NP_112391.1 |
| Canis^a^ | NCBI ID | XP_536998.1 |
| Drosophila | NCBI ID | NP_524416.1 |
| Pan | GenBank | JAA13745.1 |
| Danio | NCBI ID | NP_001136426.1 |
| Bos | GenBank | DAA15620.1 |
| Xenopus | NCBI ID | NP_001080414.1 |

a. Predicted sequence.

Figure S1. ITC studies on the Cysteine mutants

(a) The SOCS Cys mutants were titrated against EloBC. The raw data are shown on top, the heat integration at the bottom. The resulting Kd is given for each construct. (b) Thermodynamic analysis of the ITC binding assay. The binding free energy (ΔG), observed enthalpy (ΔH_obs_) and entropy (-TΔS) are plotted for the Vif fusions proteins binding to EloBC.

Figure S2. Amino acid sequence alignments of Vif and EloB

(a) The conservation of the Vif PPLPS motif in different HIV-1 strains. The BC-Box and the proline rich motif in Vif sequence are highlighted with underscore. (b) The conservation of the DVMK stretch in EloB homologous proteins. The DVMK stretch in EloB is highlighted in red.

Figure S3. ITC raw data of the SOCS-EloBC binding studies

The raw data are shown on top, the heat integration at the bottom.
